# Tips and Tricks of Aortic Valve Reimplantation to Treat Aortic Root Aneurysms

**DOI:** 10.21470/1678-9741-2026-0072

**Published:** 2026-11-01

**Authors:** Tirone E. David

**Affiliations:** 1 Division of Cardiovascular Surgery, Toronto General Hospital, Peter Munk Cardiac Centre, University Health Network, University of Toronto, Toronto, Ontario, Canada

**Keywords:** Aortic Valve Reimplantation, Aortic Root Aneurysm.

## Abstract

This article summarizes the author’s experience with aortic valve reimplantation, focusing on technical errors that may adversely affect outcome. This is a complex surgical procedure that requires detailed knowledge of the functional anatomy of the aortic valve and how disease affects its components. The procedure was developed to correct abnormal components of the aortic root in patients with aortic root aneurysms, both sporadic and associated with genetic syndromes, with the aim of restoring the native aortic valve anatomy and function. When applied to properly selected patients and performed correctly, the very long-term outcomes are gratifying for both the patient and the surgeon.

## INTRODUCTION

Echocardiography plays a central role in assessing the functional anatomy of the aortic valve in patients with aortic root aneurysms and in selecting suitable patients for aortic valve reimplantation. It is also indispensable in the operating theater. Preoperative assessment of aortic valve morphology and function is the first step in determining whether the aortic valve can be preserved. Each component of the aortic root must be identified and visualized in multiple views. The appearance and diameter of the left ventricular outflow tract and aortic annulus, the distance between the aortic and mitral valves (intervalvular fibrous body), the aortic cusps, and the sinotubular junction must be interrogated from every angle. Whether tricuspid or bicuspid valves, it is important to visualize the aortic cusps to assess their thickness, mobility, and structural integrity. Lemaire and Vancraeynest^[1]^ have developed a didactic method for aortic root assessment in six steps: 1. determine the aortic valve phenotype; 2. determine the aortic root phenotype; 3. assess the aortic valve function; 4. assess leaflet tissue quality; 5. measure all components of the aortic root; 6. look beyond the aortic valve and the entire heart and great vessels.

## OPERATIVE TECHNIQUE AND COMMON PITFALLS

A detailed description of the operative technique for aortic valve reimplantation, as performed by the author, was recently published^[2]^.

### Cardiopulmonary Bypass and Myocardial Protection

Aortic valve reimplantation is performed through a median sternotomy (partial or complete) and under cardiopulmonary bypass. This is a long operation, and myocardial protection is of utmost importance. I have used blood cardioplegia during the past four decades, and impaired ventricular function due to inadequate myocardial protection has just about been abolished. I deliver cardioplegia directly into the coronary arteries through soft-tip cannulas, which are secured to the aortic sinus next to the coronary artery orifice. The perfusionist is instructed to administer an initial dose of 1 liter of high-K+ blood cardioplegia, followed by 500 mL every 20 to 30 minutes at 150 to 250 mL/min, depending on heart size and myocardial thickness.

#### Pitfalls

1. The cardioplegia cannula can damage the intima of the coronary artery orifice. Do your best to prevent complication by using soft-tip cannulas smaller than the coronary arteries and insert and handle them with care. If it is obvious that an intimal tear occurred at the origin of the artery, open it and repair with a vein patch under direct vision, or use a vein graft to bypass it beyond the intimal tear. In doubt, and if the patient is stable, transfer to the cardiac catheterization laboratory and get coronary angiography. The interventionist may correct the problem with a stent if the tear or dissection is localized. If it is more extensive, you have to return to the operating room and bypass the dissected or occluded artery.
2. Patients with bicuspid aortic valves frequently have a dominant circumflex artery and a short or no left main coronary artery, and cardioplegia may not be delivered to both the left anterior descending and the circumflex arteries. One may use two smaller cannulas, one for each coronary artery. Additionally, the non-dominant right coronary artery may be too small for direct cannulation to deliver cardioplegia, and a hand-held cannula may be needed. An unprotected right ventricular free wall can turn into a postoperative disaster.

### Preparing the Aortic Root for the Aortic Valve Reimplantation

After transecting the ascending aorta 1 cm above the sinotubular junction and starting to protect the heart with antegrade cardioplegia, the aortic valve cusps are carefully examined to determine that they are suitable for preservation. The aortic cusps are, after all, the most important components of the aortic valve. The cusps must be pliable and free of calcification. They must have some residual elasticity (rather than being sclerotic and inelastic), and in my experience, the more elastic they are, the better the long-term durability inside a rigid tubular Dacron® graft.

The coronary aortic sinuses are excised, leaving 4 - 5 mm of sinus tissue attached to the aortic annulus. Detach the coronary arteries and use stay sutures on the drapes to keep them away from the aortic valve. Next, dissect the aortic root circumferentially to a level below the aortic annulus by at least 2 - 3 mm, starting at the aorto-pulmonary ligament, as if you were doing a Ross procedure. This area allows for the separation of the right ventricle from the interventricular septum, but as the dissection progresses toward the right atrium, the right ventricle may be accidently open. Do not attempt to close the tear in the right ventricle at this stage. The right atrium is often attached at a mid-level of the membranous septum, and it should not be detached from its insertion. The root can be easily freed from the dome of the left atrium, but if its insertion is at the level of the aortic annulus, avoid entering the left atrium.

#### Pitfalls

One may accidentally enter the right ventricular cavity, and air may enter the venous line. Control the air by snaring the cava if bicaval cannulation was used or by placing a saline-soaked sponge on the right ventricular wall. Avoid entering the right atrium insertion in the aortic root at the level of the membranous septum. Avoid entering the left atrium if attached at the level of the aortic annulus. Repair of right ventricular tear is performed after the graft is secured to the outflow tract. A simple running suture between the right ventricular wall and the Dacron® graft resolves the problem.

### Placing the Left Ventricular Outflow Tract Sutures

Place at least nine interrupted sutures of 2-0 polyester with soft felt pledgets along a single horizontal plane except in the area of the membranous septum where the sutures should be passed through the membranous septum and right atrial wall attached to its external wall. Larger aortic annulus (*e.g.* > 30 mm in diameter) requires more sutures, particularly along the connective tissue parts of the left ventricular outflow tract. If parts of the left atrial wall could not be separated from the aortic root, the annuloplasty sutures should incorporate the left atrial wall.

#### Pitfalls

Passing the sutures in the junction of the membranous septum with the muscular septum may injure the Bundle of His and cause heart block. If one is uncertain where the Bundle of His is located, avoid suturing in that area and leave a 6- to 8-mm gap without sutures, that is, in between the membranous septum and the muscular septum junction. Depending on the rotation of the left ventricle in relation to the aortic root in patients with bicuspid aortic valves, there may be no obvious membranous septum, and the determination of where to place the sutures is more difficult to avoid the Bundle. If there is no membranous septum, I leave a 6 - 8 mm gap in the area where the Bundle normally lies.

### Choosing the Size of the Dacron® Graft and Securing it to the Outside of the Outflow Tract

I continue to believe that the average height of the cusps is the best and most reliable method to estimate the size of the graft^[3]^. However, I always measure the diameter of the aortic annulus as well as the diameter of the sinotubular junction by pulling the three commissures upwards and observing the distance among the commissures that allows the cusps to coapt centrally. If in doubt between two sizes, always take the larger one. Over the past two decades, we have used a graft one size larger than estimated and plicated the intercommissural spaces along the sinotubular junction to reduce the graft diameter after reimplantation of the aortic valve, thereby creating neo-aortic sinuses.

The spacing of the sutures as they are passed through the end of the graft is crucial for properly reducing the diameter of the aortic annulus in patients with annuloaortic ectasia. The sutures must be spaced radially from the center of the aortic root towards the graft. A large annulus means that the sub-commissural triangles are widened, and these are the areas that should be reduced by placing the sutures closer together in the graft than they are in the outflow tract. Next, the sutures are tied on the outside of the graft. The assistant should pull the three commissures upward while the surgeon ties the annuloplasty sutures to ensure the left ventricular outflow tract lies within the graft.

#### Pitfalls

The entire aortic valve and its annulus must be inside the graft by at least 5 - 6 mm above the suture line. If this does not occur after the sutures are tied on the outside, it means that the circumferential dissection of the aortic root was not deep enough, or the sutures were placed too high in the outflow tract, or the distribution of radial sutures was incorrect. It is better to take down what has been done and restart this part of the operation. Placing the entire aortic valve inside the tubular structure is crucial for a durable reconstruction of the aortic root.

### Resuspension of the Commissures Inside the Dacron® Graft

The three commissures should be pulled upwards and temporarily secured to the graft with a transfixing polypropylene suture with a small Teflon® felt pledget. Ideally, the three commissures should be at the same level, or at least the commissures of the non-coronary aortic cusp must be at the same level. This can be verified by examining the graft's corrugation. The graft does not have to be pulled to straighten its corrugation. What is important is that the level of the three commissures be approximately the same within the graft. The commissure between the left and right cusps is often shorter than the other two in patients with aortic root aneurysms. The level of the commissures within the graft and the space among them must be based on the size of the aortic cusp. As a rule, the left cusp is smaller than the other two, and its commissures should be closer together than the other two. Next, the aortic annulus must be secured on the graft. This should be done with transfixing sutures by successively passing through the junction between the annulus and the remnant of the aortic sinus and bringing back inside the graft and passing through the remnant of the sinus wall, 3 - 4 mm apart and in between bites. One should not run the sutures from within the graft because it may distort the annulus. Transfixing sutures, as described above, ensures that the annulus is attached to the graft.

#### Pitfalls

Incorrectly suspended commissures will affect the cusp morphology and motion. Thus, before starting to secure the remnants of the sinus wall in the graft, make absolutely certain that the level of the commissures is spatially correct.

### Adjusting the Height of the Aortic Cusps

The aortic cusps must coapt centrally and well above the nadir of the aortic annulus. In approximately one-half of my patients, one or more cusps were prolapsing and required plication of the free margin along the nodule of Arantius. If a cusp has a fenestration close to the commissure and is being held by a strand of cusp tissue, I firmly believe that the free margin should be reinforced by weaving a double layer of a fine expanded polytetrafluoroethylene suture along the free margin, 1 - 2 mm apart, and 1 mm below each other.

Although I have never used it, Schafers’ caliper for determining the height of the cusp after reconstruction of the aortic root is considered useful by many surgeons. The Dacron® graft is so stiff that it is not difficult to examine the free margins of the cusps and visually determine where they stand within the aortic root and in relation with each other.

#### Pitfalls

Determining the level at which the aortic cusps will coapt after reimplantation of the aortic valve may be difficult for the inexperienced surgeon, and perhaps the Schafers’ caliper should be used. Unfortunately, the final result will only be seen after discontinuation of cardiopulmonary bypass and echocardiographic examination of the aortic valve function. Cusp prolapse is the most common cause of a second pump run after this operation. Trace or mild aortic insufficiency with good geometric alignment of the cusps is acceptable, but if the coaptation height or length is inadequate, even mild aortic insufficiency will progress to moderate and severe, and eventually reoperation.

### Reimplantation of the Coronary Arteries and Creation of Neo-aortic Sinuses

Most surgeons have no difficulty in reimplanting the left main coronary artery, but even experienced ones may have problems with the right coronary artery. A common practice is to distend the right ventricle and determine where the artery should be reimplanted. My personal preference is to take a mental photograph of the right coronary artery's orifice relative to the aortic annulus and commissures before detaching this artery from the aortic root aneurysm. It is then reimplanted at the same level within the graft, which is often as high as the sinotubular junction and slightly toward the commissure between the left and right cusps. Unfortunately, only after discontinuation of cardiopulmonary bypass can one be certain that the right coronary artery is properly perfusing the myocardium.

I believe that the inter-commissural spaces should be plicated to create neo-aortic sinuses. Alternatively, a graft one size smaller can be sutured to the graft used for aortic valve reimplantation and anastomosed at the level of the new sinotubular junction.

#### Pitfalls

As mentioned above, reimplantation of the right coronary artery can be troublesome, and it does not become apparent until several minutes after discontinuation of cardiopulmonary bypass and echocardiographic evidence of right ventricular and posterior wall dysfunction. After excluding air embolism, I inspect the reimplantation site and the proximal right coronary artery for a kink or fibrous band that may pull on the artery and distort blood flow. If ventricular function does not improve, the safest course is to return to bypass and revise the reimplantation side. Most patients are fairly young, and I prefer to detach the artery, patch the hole with a piece of Dacron® graft, and reimplant the artery in an area that is not likely to cause interference with blood flow once the right ventricle is distended. Here again, the operating room is the best place to address these problems. Once they are transferred to the intensive care unit, it is far more difficult to correct technical problems.

## FINAL COMMENTS

The more complex the operative procedure, the more likely we, as surgeons, are to create new pathologies that affect the outcome of the intended procedure. Attention to detail and careful dissection, suturing, and alignment of the anatomic structures are indispensable to perform aortic valve reimplantation. The reward of a well-executed operation is priceless for both the patient and the surgeon.

## Data Availability

The data supporting the findings of this study are owned by Dr. Tirone E. David’s Clinic and were used under license for this research. Due to contractual restrictions and ethical and legal requirements for protecting patient data, the datasets cannot be made publicly available.

## Artificial Intelligence Usage

The authors declare that no artificial intelligence tool was used in the preparation of this article.
